# Co-Created Digital Pretherapy Psychoeducation for Outpatients in Specialized Mental Health Care: Usability Evaluation and Patient Satisfaction Study

**DOI:** 10.2196/80130

**Published:** 2026-02-26

**Authors:** Henrik Pedersen, Tatiana Skliarova, Liv S Engvik, Arthur Mandahl, Carlos De las Cuevas, Audun Havnen, Mariela Loreto Lara-Cabrera

**Affiliations:** 1 Nidaros DPS St Olav's University Hospital Trondheim Norway; 2 Department of Mental Health Norwegian University of Science and Technology Trondhem Norway; 3 Vårres Regional User-Led Center Trondheim Norway; 4 Department of Internal Medicine, Dermatology and Psychiatry Universidad de La Laguna La Laguna Spain; 5 Department of Psychology Norwegian University of Science and Technology Trondheim Norway; 6 Nidelv DPS St Olav's University Hospital Trondheim Norway

**Keywords:** usability testing, mental health, mental disorder, patient-centered care, internet-delivered, patient satisfaction, usability, digital health, patient and public involvement

## Abstract

**Background:**

Specialized mental health services are facing high demand, potentially leading to lower-quality care. One solution may be to prepare patients for attending treatment. Digital pretherapy psychoeducation may be particularly relevant. However, the effectiveness of such an intervention depends on user engagement and satisfaction, and usability is therefore one of the most important factors.

**Objective:**

This article has 2 objectives. Study 1 describes the development of StartHelp, a co-created digital pretherapy psychoeducation program for patients on waiting lists before their first consultation in outpatient specialized mental health services. Study 2 explores the usability of StartHelp, aiming to identify potential issues and assess whether the intervention is suitable for further evaluation in a randomized controlled trial.

**Methods:**

Guided by co-creation principles, we developed StartHelp in accordance with the Guidance for Reporting Involvement of Patients and the Public (GRIPP2) checklist. To assess the app’s usability, we recruited 10 patients from specialized mental health care to complete tasks during individual think-aloud interviews. Afterward, they completed questionnaires, including open-ended questions, an item assessing perceived video quality, 2 versions of the System Usability Scale (SUS), the 4-item Client Satisfaction Questionnaire (CSQ-4), and a modified version of the CSQ-4 (CSQ-Video). The StartHelp project group discussed the results, and solutions to the identified issues were proposed and implemented.

**Results:**

Study 1 involved the development of StartHelp over 24 months. The app comprises 27 tasks, including 24 videos and links to 14 websites containing in-depth information. Study 2 involved usability testing with 5 men and 5 women. SUS scores for StartHelp’s videos indicated good usability, with a mean of 83.7. By contrast, SUS scores related to navigating StartHelp’s overarching architecture were barely acceptable, with a mean of 63.6. For the CSQ-4, the sample mean was 12.3, indicating moderate satisfaction. Mean scores on the CSQ-Video (10.9) indicated satisfaction in the lower moderate range. However, patients perceived the videos as high quality and rated them as nonoffensive. The qualitative findings supported the quantitative results. The usability tests revealed 1 major issue and several minor issues. The primary issue concerned navigation of the overarching technological infrastructure on which StartHelp was developed, rather than StartHelp itself. To address these issues and impracticalities related to interacting with the overarching technological infrastructure, we made minor changes to the StartHelp app.

**Conclusions:**

Through a collaborative co-creation process, we developed StartHelp, a digital pretherapy psychoeducation program. Usability testing indicated that the content itself was highly usable, with video-related SUS scores suggesting good usability, whereas navigation of the overarching technological infrastructure received lower scores. Although patients rated the clinical material as high quality and nonoffensive, satisfaction scores were lower than anticipated. Nevertheless, StartHelp was deemed ready for further testing in a clinical trial.

## Introduction

### Background

Globally, the high prevalence of mental disorders represents a growing public health concern [1,2]. This prevalence leads to heightened barriers to care in specialized mental health services [2] and results in a decline in service quality due to the measures implemented to address the high demand [3]. Such measures may include more restricted services or longer waiting times before the initial consultation, which could result in unmet needs [2], lower attendance [3], worsening of mental health symptoms during the waiting period [3], or changes in patient-reported variables that predict positive outcomes in future therapy [4]. To address this issue, a pluralistic approach is likely necessary, with a simultaneous focus on traditional psychotherapy, adjunctive interventions, and innovative technology-assisted approaches [2,3,5,6].

One type of intervention that may help meet this demand is the preparation of patients for specialized mental health services through psychoeducation [7-11]. Such pretherapy interventions have demonstrated promising results by increasing patients’ knowledge of treatment options [9]. Furthermore, improvements in patient satisfaction [12] and patient-reported outcomes [7,12-14], reported in both the short term [8] and long term, suggest an association with more favorable treatment outcomes [7,8].

One dilemma faced by pretherapy interventions is the allocation of therapist resources: specifically, whether therapists should dedicate their time to preparing or treating patients. One way to address this issue is to involve user representatives as close collaborators, delivering pretherapy interventions in a group format [7-9], thereby reducing the number of health personnel required. Another solution is to provide pretherapy psychoeducation digitally [10], particularly given its potential for scalability [6,15,16] and lower barriers to entry [10]. However, researchers have highlighted the lack of rigorous testing of pretherapy digital interventions [10], both during their development and in clinical trials [15,16]. Some studies have also identified adverse effects of digital pretherapy [10], indicating that preparation is not without risk and that thorough evaluation is crucial. Moreover, the outcomes of digital interventions are highly dependent on user engagement and satisfaction, with usability being the primary barrier to engagement [17]. In other words, usefulness, ease of use, and satisfaction are core components of usability [18].

Recent developments have underscored the importance of patient and public involvement in research [19,20]. This involvement can begin during the development of health interventions [20-22], a practice commonly referred to as “co-creation” [22-24]. The process of co-creation goes beyond gathering user feedback; it involves active collaboration with all relevant nonacademic stakeholders throughout the research and development phases, grounded in a patient-centered perspective [25]. This approach results in more relevant content, increased engagement, and ultimately more effective implementation [20,22-24,26]. Despite these potential benefits, however, patients and user representatives are rarely engaged in the co-creation of mental health interventions [22], including both digital and nondigital pretherapy interventions [10,26].

### StartHelp

To address these gaps in the literature, we developed StartHelp (Norwegian: “StartHjelp”), a co-created digital pretherapy psychoeducation program for patients awaiting their first consultation at outpatient specialized mental health services. StartHelp is designed to address the gap between referral and a patient’s initial appointment with a therapist. The primary goal in developing StartHelp was to provide patients with essential information before starting treatment in a digital, automated way. This information includes legally mandated information, such as patient rights, as well as details regarding the psychological assessment process, practical considerations, and coping strategies during the waiting period. Moreover, different treatment approaches are presented, including video consultations, group therapy, therapist-guided internet-based interventions, medical treatment, and physical therapy. The aim of providing this information to patients before their first consultation is to enhance patient-centered care by preparing them for their upcoming therapy. Within this framework, StartHelp aims to improve patient satisfaction by making the waiting period more meaningful, clarifying the patient’s role in therapy, establishing realistic expectations, and alleviating apprehension about the initial sessions.

### Objectives and Hypotheses

This study aimed to evaluate whether StartHelp was ready for real-world testing in a clinical trial and had 2 objectives. The first was to describe the development process of StartHelp (study 1). The second objective was to investigate its usability by assessing program navigation, potential adverse effects, and suggestions for improving 4 of StartHelp’s videos (study 2). We hypothesized that StartHelp would achieve a System Usability Scale (SUS) score of >70, based on benchmark values for digital health apps [27] and Scandinavian patient portals [28]. Additionally, we hypothesized that StartHelp would achieve medium levels of satisfaction.

## Methods

### Study 1: Presentation of StartHelp and Its Development

#### Overview

We used the Guidance for Reporting Involvement of Patients and the Public (GRIPP2) checklist [19] as a reporting framework to ensure transparent reporting of user involvement (see Multimedia Appendix 1).

#### Ethics Approval

All necessary ethical approvals were obtained before the study began. The study protocol and consent form were evaluated and approved by the Regional Committee for Medical Research Ethics in Mid Norway (*Regional etisk komite* [regional ethics committee]: 2024/729793) and the Norwegian Agency for Shared Services in Education and Research (Norwegian: Kunnskapssektorens tjenesteleverandør [SIKT]: 2024/987092). When initially contacted, patients were told that participation was independent of their treatment process and that their decision whether to participate would not affect their treatment in any way. They were also informed that they could send an SMS text message at any time to cancel the appointment without having to explain why. Patients formally provided informed consent by signing a consent form on-site before starting the testing. The form consisted of information about the study, how the data were handled to secure anonymity, and patients’ right to opt out of the study at any time without any consequences for treatment.

#### Description of StartHelp

StartHelp is a mobile app accessed through the official regional patient portal [29]. In StartHelp, patients are prompted with tasks at predetermined intervals, consisting of psychoeducation delivered through text and video. This stepwise approach serves 2 purposes. First, we believe that delivering information in this manner ensures that patients are not overwhelmed, allowing them to review the information carefully. Second, we reasoned that this approach may promote ongoing engagement and foster a sense of continuity during patients’ time on the waiting list.

**Figure 1:**
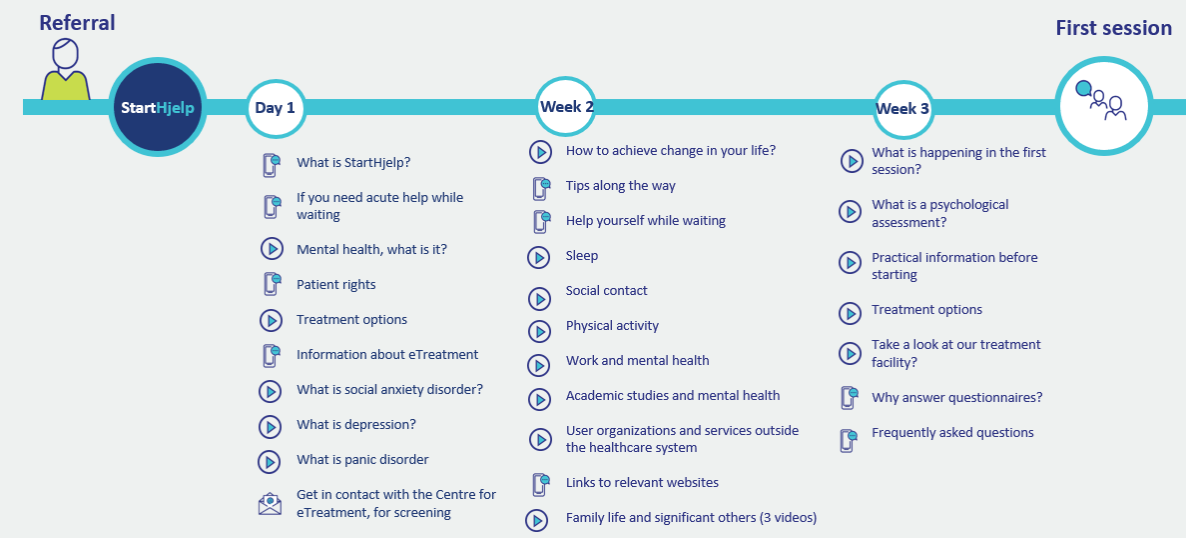
StartHelp content and timeline.

**Figure 2:**
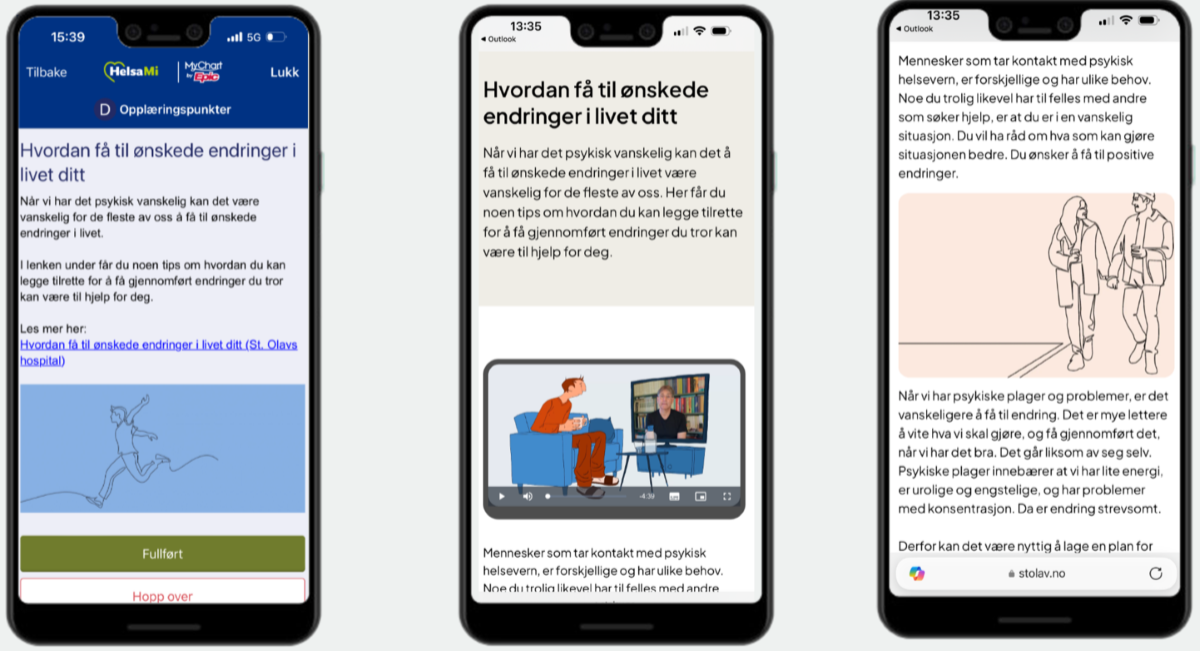
Visual presentation of tasks in StartHelp.

**Figure 3:**
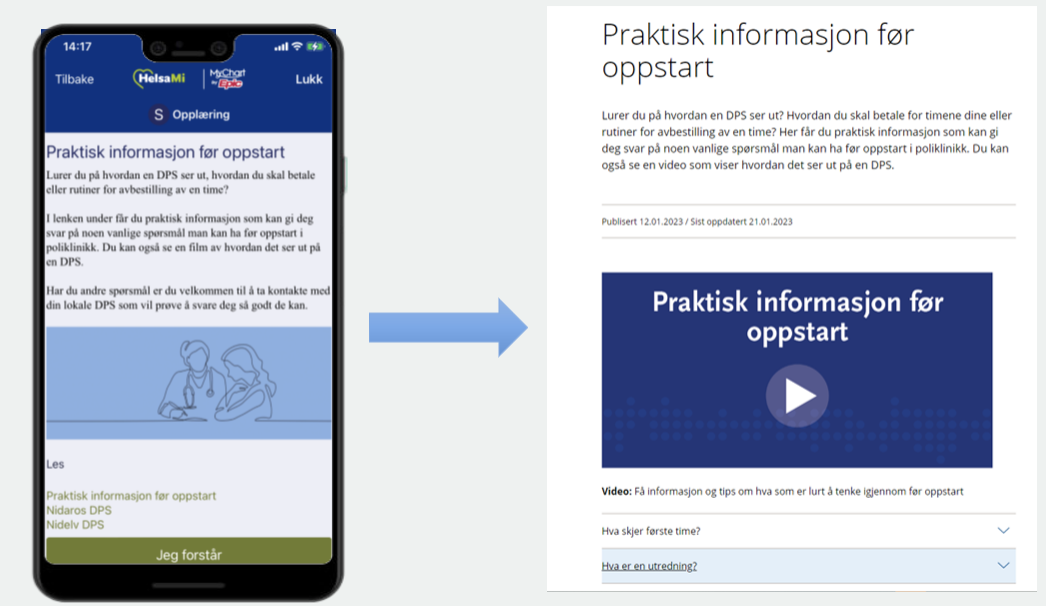
Visual presentation of a task and website with in-depth information.

**Figure 4:**
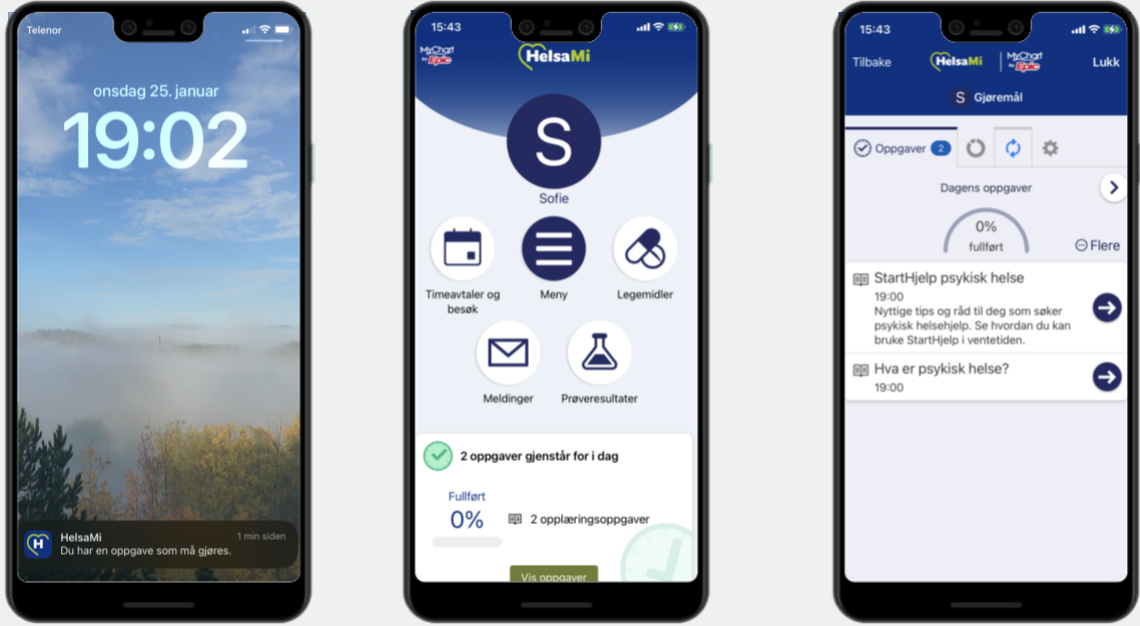
Visual presentation of phone notifications, the MyHealth front page, and StartHelp dashboard.

StartHelp’s dashboard consists of a list of tasks that automatically updates. Clicking on a task reveals a brief text consisting of 1 or 2 paragraphs, and most tasks provide hyperlinks to websites containing more in-depth information [30]. The destinations of these links include websites of official Norwegian health authorities, patient organizations, official legal authorities, and “closed-off” websites hosted by the university hospital and created by the project group. These websites are accessible only through the app and not via search engines. On these websites, short videos accompanied by text provide more detailed information. The videos range in length from 1 to 3 minutes, with 1 video exceeding 4 minutes. In total, StartHelp consists of 27 tasks, 14 websites, and 24 videos. Figure 1 presents an overview of the task content and the timing of task administration. Figures 2 and 3 show screenshots of StartHelp and a website with a video and accompanying text. Figure 4 depicts the StartHelp dashboard in its right frame.

The intention was for every patient to read the initial information and watch most of the videos before selecting the in-depth information relevant to them. As patients must actively press a button to mark a task as complete, the app can track their activity, and checkmarks indicate which tasks have been completed.

#### Description of the Overarching Technological Infrastructure

StartHelp is not a standalone app; it is built upon an overarching technological infrastructure known as MyHealth (Norwegian: HelsaMi). MyHealth serves as a patient portal connected to the regional patient administration and journal system in the Central Norway health region. Patients need MyHealth to access StartHelp. StartHelp is free to use and does not require user registration.

MyHealth was designed to provide patients and, occasionally, their relatives with easy access to patient information and health care services. It offers a range of functions, including completing questionnaires relevant to the patient’s care; providing secure video consultations; notifying the health care provider of the patient’s arrival at the clinic; posing questions to the health care provider; receiving information; and providing an overview of planned consultations, personal information, medical prescriptions, and journal entries. Figure 4 presents screenshots of MyHealth and StartHelp, with the MyHealth front page displayed in the middle. MyHealth is comparable to other patient portals in other health regions across Norway and other Nordic countries due to collaborations with the same health system providers [28,31,32].

In addition to serving as a patient portal, one of MyHealth’s goals is to provide a secure infrastructure integrated into the regional journal system, enabling the development of additional innovative programs tailored to specific patient groups without involving private third-party actors.

#### Development of StartHelp

##### User Involvement and Co-Creation

User involvement was deemed essential from the beginning of the development process, both in principle and to ensure the quality, relevance, and implementation of the final product [20,22]. As the objective was to digitize prior pretherapy educational interventions [7-9], user representatives who had collaborated in their design and delivery were contacted. We strove to ensure that these user representatives held a partner role, meaning they would have equal influence with other members of the project group in making important decisions throughout the process [33]. In addition to user representatives, the project group included experienced nonacademic clinicians, psychologists, psychiatrists, psychiatric nurses, and clinic middle management to ensure that all relevant perspectives were represented [34].

First, the group reviewed previous interventions and current service needs. User representatives highlighted frustrations regarding long wait times and limited communication. Consequently, the team identified a critical need for information regarding treatment options, self-help, patient rights, and user organizations, with a preference for video content over written text.

##### Development of Video Manuscripts and Informational Websites

The project group decided to develop 2 general types of videos for StartHelp: one featuring a clinician speaking directly to the camera, accompanied by visual animations illustrating key points (experts talking), and animated videos with a voice-over.

Most of the work on the manuscripts for these videos was conducted collaboratively with the project group via videoconference. We collectively outlined the objectives for each video, aiming for concision. Thus, patients who find the content relevant may feel encouraged to explore the in-depth information provided through the links. The intention was that this proactive method of seeking relevant information would also engage patients at an early stage. We then allocated responsibility for the manuscripts among the project group members and revised them together in subsequent meetings. In this process, the user representatives exerted significant influence on the phrasing.

After completing the video manuscripts, the project group contacted an advertising agency with an existing contractual relationship with the university hospital. The agency filmed all the videos, produced the animations, and recorded the voice-overs, which underwent iterative testing in 2 workshops with 6 participants each. Participants, recruited by user representatives, included individuals with attention-deficit/hyperactivity disorder, autism spectrum disorder, or both. This approach helped ensure that the content was accessible and comprehensible to a broad audience.

The feedback that emerged from the workshops prompted significant design changes. Participants rejected an initial “cartoon character” style as too similar to commercials and too distracting. We adopted a “1-line drawing” style that gradually appears on screen and decided to maintain this graphic style throughout StartHelp. Based on user requests, the project group also created an additional introductory video.

##### Website Design

After finalizing the video design, we created websites with comprehensive information. These websites use the hospital’s web domain, which imposed template constraints, but we maintained the videos’ style throughout. The content was designed for easy scanning, using headlines that function as drop-down menus.

**Figure 5:**
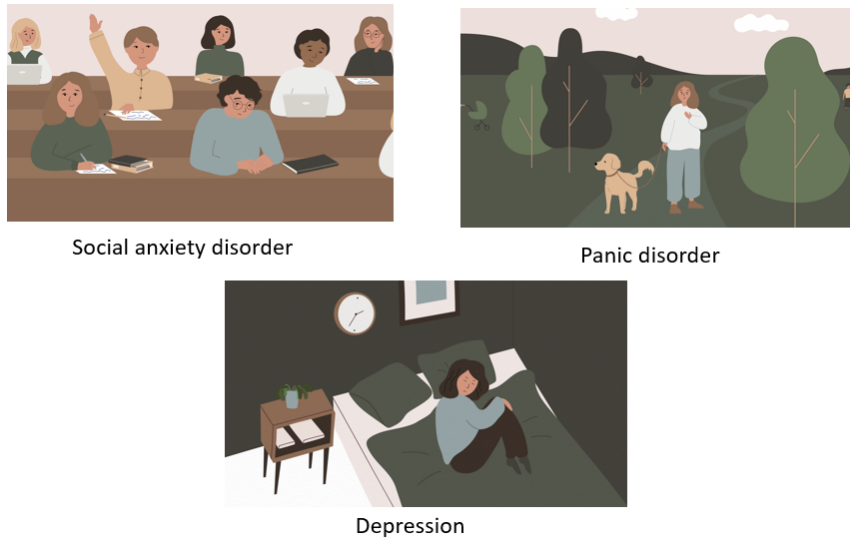
The graphic profile of the videos depicting specific mental issues.

During the development of the websites, the project group realized the need for 3 additional videos addressing panic disorder, social anxiety, and depression. The videos aimed to lower barriers to entry for specific treatment options, such as therapist-led internet treatment and group therapy. The videos used composite narratives derived from interviews with 3 patients per diagnosis to ensure anonymity and relatability. Unlike the earlier content, these videos were not tested in workshops and were therefore included in the usability testing. The graphic profile of these videos is illustrated in Figure 5.

##### Initial Paper Testing

In January 2023, the project group conducted a “paper test” of a StartHelp prototype before integrating it into MyHealth. The group outlined the prototype in Miro Board, a cloud-based interactive whiteboard. The visual drafts were printed on paper and tested on 4 individuals aged 25-64 who were recruited from the associated outpatient mental health clinic.

**Figure 6:**
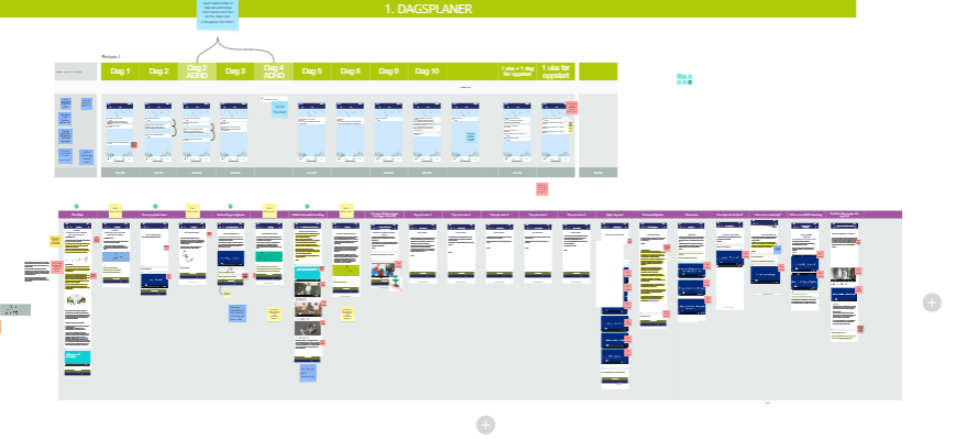
Visualization of paper prototype testing.

In this test, patients navigated back and forth within the paper-based prototype app. They described which buttons they would press, and a flowchart guided them to different pages in the paper prototype. Two members of the project group participated in each user test: 1 conducted the test, and the other took notes based on the users’ feedback. The test produced 10 key insights. These insights were then categorized according to recurring themes. Figure 6 depicts this process.

Finally, the project group conducted preliminary checks for major technical bugs before planning and conducting the usability testing outlined in study 2.

### Study 2: Usability Testing

#### Methods and Procedures

Study 2 explored the usability of StartHelp, aiming to clarify usability issues that may hinder a clinical trial. Usability testing was conducted between May and July 2024 using a convenience sample of patients receiving treatment at a Norwegian outpatient mental health clinic. These patients were contacted because StartHelp’s user base will primarily consist of individuals from the general clinical mental health population. Although StartHelp is intended for patients who have not yet started treatment, we recruited patients who had attended at least one consultation.

This approach ensured that participants had prior experience with the MyHealth patient portal, facilitating differentiation between StartHelp itself and the overarching portal infrastructure, MyHealth. We considered that this distinction would have been more difficult if both systems had been introduced simultaneously for the first time. During usability testing, the facilitator explicitly presented StartHelp as a distinct program accessed within MyHealth, and participants were instructed to evaluate StartHelp separately from the patient portal.

Research on usability testing has found that samples of 5 participants typically identify approximately 80% of usability issues [35]. To increase the likelihood of identifying not only common but also less frequent and potentially critical usability issues, we chose to include 10 participants. A sample of this size is considered sufficient to approach thematic saturation in qualitative usability data and to capture a broad range of user experiences relevant to refining the intervention before evaluation in a clinical trial. As recommended by Maramba et al [36], both qualitative and quantitative data were collected and interpreted together. This approach enables a comprehensive assessment of usability while highlighting specific issues that require attention.

To recruit patients for testing, the first author discussed the project with clinicians at a community mental health center in central Norway. There were no formal exclusion criteria other than requiring acute care due to suicide risk, current psychosis, or an inability to provide informed consent. Clinicians then asked patients whether they would be interested in learning more about a research project concerning a digital pretherapy intervention. If patients expressed interest and consented to be contacted, the first author (HP) called them, explained the project details (eg, the testing goals and expected duration), and scheduled a date and time for testing.

#### Data Collection and Analysis

The individual testing comprised 3 phases and lasted between 45 minutes and 1 hour in total. First, patients completed a series of questionnaires; afterward, they were assigned a set of tasks that included watching 4 selected videos before completing another set of questionnaires. At the end, they were asked the open-ended question, “Do you think all relevant issues regarding the app you tested today have been addressed in our conversation and the open questions in the questionnaires?” The think-aloud interviews were conducted by a facilitator (HP) who received training in the think-aloud methodology, including guidance on using neutral prompts and minimizing leading questions to reduce potential interactional bias. A member of the research team was present throughout all phases to observe and provide assistance. If participants had any questions, the facilitator responded with standard phrases such as “What do you think?” “What would you do?” “Are there any ambiguities we should become aware of?” or “If you find it difficult to circle an answer, please circle the response that you first thought of.”

#### Tasks

After answering the first set of questions, participants were instructed to complete a series of tasks. The instructions provided to participants before starting are presented in Textbox 1. The tasks themselves are presented in Textbox 2. During task completion, the concurrent think-aloud method was used [37]. The facilitator observed all participants during the tasks but sat at a desk elsewhere in the room while they completed the questionnaires.

**Instructions given orally to participants before starting on tasks.**

- “Now, you will receive a series of tasks from me. These tasks will be quite straightforward and will involve navigating the program called StartHelp. The purpose of this exercise is to determine whether the program is easy to use.”
- “The tasks may be easy for you to complete, but if they prove challenging, this information is valuable to us. Only by identifying what is difficult or illogical and trying to understand why can we determine what improvements are necessary. Therefore, we encourage you to think out loud while you solve the tasks. If, for example, I ask you to find a video titled ‘social phobia’, try your best while talking about your thought process.”
- “If at any point you find yourself stuck or uncertain about what to do, let me know. I will take note of what was difficult and assist you before moving on to the next task.”
- “I will be recording what you say so I can later write it down. This will help me to better remember everything that is said. None of what we do today will be stored with names, and the audio file will be deleted as soon as I have transcribed it. I will let you know when I have started the recording, and the first thing I would like you to do at that point is to confirm that you are aware I am recording, with a simple yes. After that, we will proceed with the tasks.”
- “While we carry out the tasks, you will also see some of the videos we have created for the final program. We have selected these videos specifically because we want your feedback. After the tasks, you will be asked what you think about them.”
- “Let me know after you have completed each of the tasks.”

**Tasks given to participants during testing.**

- “I have activated the StartHelp program for you. The first thing I would like you to check is whether you have received any form of SMS text message or email regarding this.”
- “Then, view this notification and open StartHelp.”
- “When you are inside the patient portal, the first thing I want you to visit is a page called ‘StartHelp mental health’. When you are on this page, please read the information provided. After that, you may press ‘Complete task’.”
- “Next, I would like you to locate a page that provides information about various treatment options. Watch the video on this page.”
- “After this, navigate around the same page and see if you can: (1) Find a video discussing various treatment options and watch it. (2) Find a video about social anxiety disorder and watch it. (3) Find a video about panic disorder and watch it. (4) Find a video about depression and watch it.”
- “After this, you can return to the app and ‘complete the task’.”
- “Now you can completely exit the app, returning to your phone’s home screen. After that, attempt to access StartHelp again.”
- “Now, I would like you to locate a page that says something about patient rights and click on the link provided. Afterward, I want you to try to get back to the app.”
- “Then, I would like you to navigate back to the social phobia video so that you can watch it again.”
- “If this is difficult, please return to the ‘Welcome to StartHelp’ page, where you can find instructions on how to locate it.”
- “Now, I would like you to return and locate a page that contains information about ‘e-treatment’, read the content provided there, and click the button to contact ‘The centre for eTreatment’.”

#### Videos Tested

Participants were shown 4 specific videos during task completion that the project group had determined required a more comprehensive evaluation. One video presented various treatment options available at the outpatient clinic, including face-to-face therapy (both individual and group therapy), video teletherapy, therapist-guided internet cognitive behavioral therapy, pharmacotherapy, and psychomotor physiotherapy. This video aimed to raise patients’ awareness of different treatment options and to encourage discussions with their therapist regarding treatment preferences while assessing treatment needs. The graphic profile of this video is presented in Figure 3. The other 3 videos addressed challenges associated with specific common diagnoses, such as social phobia, panic disorder, and depression. The graphic profile of these videos is illustrated in Figure 5.

These 4 videos, in particular, were tested for 2 primary reasons. First, as mentioned previously, the 3 animated videos depicting specific diagnoses had not yet been evaluated by patients or user representatives. Second, the “treatment options” video was tested because user representatives emphasized its particular importance.

#### Questionnaires

##### Demographic Variables

In addition to the aforementioned questionnaires, participants were asked to provide demographic information, including age, gender, marital status, living situation, education level, and employment status.

##### Open-Ended Questions

To further explore potential usability issues, the questionnaire included the open-ended questions presented in Textbox 3. Patients provided free-text responses.

**Open-ended questions.**

- Before completing tasks and watching videos.
  Do you know about the various treatment options available at the community mental health center? If so, which ones?
- After completing tasks and watching videos.
  Do you know about the various treatment options available at the community mental health center? If so, which ones?
  Does the MyHealth app have any specific areas for improvement?
  Do the videos you watched have any specific areas for improvement?
  Do you believe there are aspects of these videos that some patients may find condescending, offensive, or uncomfortable, either in the short or long term?

##### The System Usability Scale

The SUS [38] is one of the most widely used questionnaires for measuring the usability of digital systems and technology. It consists of 10 items, half positively phrased (eg, “I thought the system was easy to use.”) and half negatively phrased (eg, “I found the system unnecessarily complex.”). Each question is scored on a 5-point scale, ranging from 0 (strongly disagree) to 4 (strongly agree). Scores are typically multiplied by 2.5 to create an intuitive score range of 0-100 [39]. Benchmarks exist for educational tools [40], digital health apps [27], and patient portals [28] based on Scandinavian norms.

Given StartHelp’s integration with the MyHealth portal, the SUS was adapted into 2 different versions. Participants completed the SUS twice: once with MyHealth navigation and functionality in mind (SUS-MyHealth; eg, login, navigation between services; “I thought that MyHealth was easy to use”) and once with StartHelp content in mind (SUS-Videos; eg, clarity, structure, and presentation of tasks and videos; “I found the videos unnecessarily complex”). This approach was used to reinforce the distinction between the overarching patient portal and the StartHelp program during evaluation.

##### Perceived Quality of the Videos and Adverse Effects

To assess perceived quality, we used a single-item scale ranging from 0 to 100, where 0 indicated *very low quality* and 100 represented the *highest possible quality.* Moreover, to assess how uncomfortable or offensive patients found the videos, we used a scale from 0 to 5, ranging from *not at all* to a *very large degree.*

##### Four-Item Client Satisfaction Questionnaire

In this study, we used 4-item Client Satisfaction Questionnaire (CSQ-4) scores to assess participants’ baseline satisfaction, not to evaluate the effectiveness of treatment or the StartHelp intervention. The CSQ-4 [41,42], a shortened version of the 8-item Client Satisfaction Questionnaire [43,44], is a 4-item questionnaire that measures overall treatment satisfaction. Each item is answered on a 4-point scale, ranging from *no, definitely not*/*quite dissatisfied* to *yes, definitely*/*very satisfied,* yielding a possible total score range of 4-16. The Norwegian translation of the CSQ-4 [42,45] has shown psychometric properties comparable to those of the English version [41]. Cutoff values are based on Larsen et al [43], where a score of 4-10.5 indicates low satisfaction, 10.6-13.5 indicates medium satisfaction, and 13.6-16 indicates high satisfaction.

##### Client Satisfaction Questionnaire-Video

In addition to the standard CSQ-4, we used a modified version to assess satisfaction with the video content in StartHelp (hereafter referred to as CSQ-Video). Items were adapted to refer specifically to the videos (eg, “In an overall, general sense, how satisfied are you with the videos you have received?”). This adapted version has not been psychometrically validated and was therefore used for exploratory purposes only, to inform further refinement of the intervention rather than to evaluate acceptability or effectiveness.

#### Quantitative and Qualitative Data Analyses

Given the small number of patients in our sample, we analyzed all quantitative measures descriptively. We interpreted quantitative data alongside qualitative data to inform refinement and assess readiness for further evaluation in a clinical trial, rather than to establish effectiveness. Qualitative data consisted of think-aloud interview transcripts collected during task completion and participants’ written responses to open-ended questions. These data were analyzed iteratively, with all comments and suggestions reviewed. Potential usability barriers were coded into categories, and comments were organized into themes [46]. Afterward, the working group responsible for the development of StartHelp discussed both the themes and categories.

Answers to questions (considering both before and after; see Textbox 3) “Do you know about the various treatment options available at the community mental health center? If so, which ones?” were first categorized and then counted. The project group organized the responses into meaningful categories: different treatment modalities and treatments that differed in ways that make a practical difference. For example, “psychoeducation” and “psychoeducation for family members” were counted as 2 separate suggestions, as the inclusion of family members was considered significant. Conversely, “talking to a psychologist” and “talking therapy” were not regarded as distinct treatment options. However, “individual psychotherapy” and “psychotherapy over video” were classified as distinct, because awareness of video consultations may lead a patient to make meaningful choices during treatment. The listings of treatment options from all patients have been translated and are included in Multimedia Appendix 2.

#### Integration of Results Into the Development Process Through Discussion

After the first author (HP) synthesized the results, they were presented to the project group for discussion. The project group examined each patient’s suggestion and issue in relation to StartHelp’s overall goals. Potential solutions were then proposed. During this process, several key questions were considered: “Is a change in StartHelp warranted?” “What is the ideal change?” and “Is a change in StartHelp possible?”

## Results

### Development Process

The development process spanned approximately 2 years, from February 12, 2021, to January 26, 2023. During this period, we created StartHelp, consisting of a total of 27 tasks. These tasks included 24 videos and links to 14 websites containing in-depth information. A discussion of the usability testing results follows the presentation of these findings.

The insights from the initial prototype testing resulted in the following themes: the MyHealth app, the information letter regarding StartHelp, the questionnaire, general concerns, tasks within StartHelp, information in MyHealth, information on hospital websites, and videos. This feedback influenced the initial integration of StartHelp into MyHealth. As StartHelp was one of the first programs integrated into MyHealth, feedback from the user tests also led to change requests concerning MyHealth’s core infrastructure, which may benefit future programs. A digital representation of the qualitative analysis process of the Post-it notes created during prototype testing and the abstracted themes is provided in Multimedia Appendix 3.

### Usability Study Results: Participants

**Figure 7:**
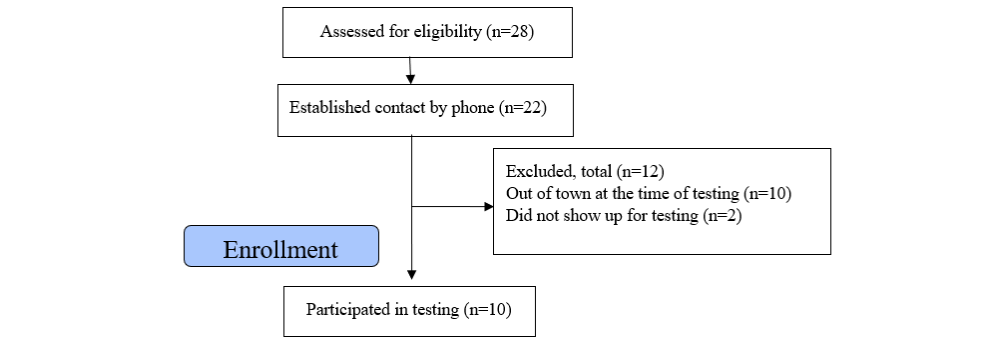
Flowchart of the usability study recruitment process.

To assess usability, we invited 28 patients to participate. Of these, 6 did not answer their phones after 2 attempts, 10 were out of town at the time of testing because it took place during the summer months, and 2 withdrew after scheduling an appointment. Recruitment concluded when 10 participants had been included. Figure 7 presents a flowchart of this process.

The mean age of the sample was 31.8 (range 21-44) years. Half of the participants (n=5) were women, and 6 of the 10 were married or cohabiting. Most (n=8) had completed 3 or more years of higher education, and 7 were on sick leave. Table 1 presents the collected demographic information for each patient.

**Table 1:** Demographic information about participants.

Patient ID	Gender; age (years)	Marital status	Living situation	Education^a^	Employment status	Work domain
Patient 1	Female; 44	Not married	Living with someone	Higher secondary education	Currently on sick leave	Management/administration
Patient 2	Female; 33	Married/cohabiting	Living with someone	Higher education—3 years or less	Currently on sick leave	Cultural work/design/music/sport
Patient 3	Male; 31	Married/cohabiting	Living with someone	Higher education—3 years or less	Currently on sick leave	Health care
Patient 4	Female; 40	Married/cohabiting	Living with someone	Higher education—5 years or more	Employed	Education
Patient 5	Female; 33	Not married	Living with someone	Higher education—3 years or less	Partial sick leave	Education
Patient 6	Male; 31	Married/cohabiting	Living with someone	Higher education—3 years or less	Currently on sick leave	Management/administration
Patient 7	Male; 26	Married/cohabiting	Living with someone	Higher education—5 years or more	Employed	Management/administration
Patient 8	Male; 23	None of the answers fit my situation	Living with someone	Higher education—3 years or less	None of the answers fit my situation	Management/administration
Patient 9	Female; 36	Married/cohabiting	Living with someone	Higher education—3 years or less	Partial sick leave	Health care
Patient 10	Male; 21	Not married	Living with someone	Higher secondary education	Currently on sick leave	Construction

^a^Education refers to the highest completed education level.

### Usability Issues Emerging From Task Completion

Overall, patients experienced few usability issues during task completion and made few suggestions for improvement, although one type of issue was mentioned by all participants. These issues primarily involved navigating the overarching app infrastructure, MyHealth, rather than StartHelp itself or the videos. Participants generally felt that too many features were presented that were not relevant to them at this time. This made the MyHealth home page difficult to navigate, especially when trying to find videos they had previously watched (n=10), which had to be accessed elsewhere than through the StartHelp dashboard after completing the related task. In the original design, tasks were automatically removed from the StartHelp dashboard shortly after completion. Other comments included that the text in MyHealth was too small (n=1) and that the “sheer number of options presented at once was overwhelming” (n=3). Finally, 3 patients had their language settings in MyHealth set to English instead of Norwegian, which made the StartHelp instructions for navigating the MyHealth menu redundant, as they referred only to the Norwegian menu.

Finally, the fourth video, which addressed depression, had not been properly uploaded. This issue was discovered during testing, and 4 patients were therefore unable to view or comment on the video. After that day of testing, the project team contacted the information technology personnel responsible, who reuploaded the video, checked all other videos, and reviewed the upload procedures to determine whether the problem was caused by a systematic error.

### Quantitative Data

#### Treatment Options

Table 2 provides an overview of the treatment options reported by patients. Several general trends emerged from the questionnaires; 4 of the 10 patients reported a greater number of available treatment options after viewing the videos, with the total number of treatment options mentioned increasing from 19 to 31. Notably, 4 patients reported knowing no treatment options before task completion. All treatment options mentioned by patients are listed in Multimedia Appendix 2.

**Table 2:** Overview of treatment options mentioned by patients.

Treatment options^a^	Frequency before videos, n	Frequency after videos, n
Individual psychotherapy	6	9
Group psychotherapy	4	7
Video consultations	1	3
Therapist-guided internet treatment	2	1
Group exposure therapy	1	1
Psychoeducation	2	2
Family-oriented psychoeducation	1	1
Physical therapy	1	2
Counseling by a vocational counselor	1	1
Pharmacotherapy	0	3
Total suggestions	19	30

^a^A complete overview of the treatment options mentioned by patients is provided in Multimedia Appendix 2.

#### System Usability Scale and the Perceived Quality of the Videos and Adverse Effects

All patients had either equal scores on the SUS-MyHealth (mean 63.6) and the SUS-Videos (mean 85) or higher scores on the SUS-Videos.

The depression video received the highest rating; however, this score may be artificially inflated, as only 6 participants viewed the video. Moreover, the 4 participants who did not view the depression video tended to rate the videos somewhat lower than the other 6. The video on treatment options received the second-highest rating.

Most patients rated the videos as not at all or only minimally offensive (mean 1.2; range 0-5). However, 1 patient rated the videos as uncomfortable or offensive “to a very large degree,” while another rated them “to some degree.” Table 3 presents an overview of all patient scores.

**Table 3:** Descriptive presentation of survey data.

Patient ID			Perceived quality of the videos tested				
	SUS^a^ MyHealth	SUS^a^ videos	Treatment options	Panic disorder	Social anxiety disorder	Depression	Feeling of discomfort when watching videos
Patient 1	47.5	87.5	100	100	100	—^b^	0
Patient 2	57.5	75	60	70	80	—	3
Patient 3	35	72	80	50	10	—	5
Patient 4	85	87.5	90	30	30	—	1
Patient 5	95	95	100	90	90	90	1
Patient 6	65	90	95	95	95	95	0
Patient 7	47.5	70	80	90	90	90	1
Patient 8	—	87.5	70	70	70	70	0
Patient 9	77.5	87.5	90	100	100	100	1
Patient 10	62.5	85	60	60	70	70	0
Sample mean	63.6^c^	83.7	82.5	75.5	73.5	85.8	1.2

^a^SUS: System Usability Scale.

^b^Not available (patients 1-4 are missing perceived quality scores on the “depression” video due to technical problems during testing. The mean for this video was therefore calculated only for patients 5-10; patient 8 did not answer the question).

^c^This number was divided by 9 due to 1 missing datapoint.

#### Satisfaction

Regarding the CSQ-4, the sample mean was 12.3, indicating medium satisfaction with treatment [43]. The score range was 9-15. The mean score on the CSQ-Video was somewhat lower (10.9), indicating satisfaction in the lower medium range [43]. The score range for the CSQ-Video was 8-14. Table 4 presents an overview of the means and SDs for all items, in addition to the total scores.

**Table 4:** Satisfaction scores.

Item	CSQ-4^a^, mean (SD)	CSQ-Video^b^, mean (SD)
Met needs	3.2 (0.63)	2.8 (0.42)
Improvement in self-efficacy	2.9 (0.88)	2.3 (0.48)
Overall satisfaction	3.3 (0.82)	3.1 (0.74)
Come back	3.3 (0.48)	2.7 (0.67)
Total score^c^	12.3 (2.26)	10.9 (1.91)

^a^CSQ-4: 4-item Client Satisfaction Questionnaire; score range 4-16.

^b^CSQ-Video: a modified version of the CSQ-4 for evaluating informational videos; score range 4-16.

^c^A score of 4-10.5 is regarded as low satisfaction, 10.6-13.5 medium satisfaction, and 13.6-16 high satisfaction.

### Qualitative Data: Usability Issues

In response to the open-ended questions regarding potential improvements, 4 patients suggested improving the MyHealth app by tidying up the menu and reducing the number of options displayed at once. Another suggestion was to keep tasks visible on the StartHelp dashboard after completion. This would eliminate the need to exit the StartHelp dashboard and navigate through the MyHealth app to locate previously completed tasks elsewhere in the menu. In the current design, tasks were automatically removed from the StartHelp dashboard upon completion and stored under “Completed tasks.”

Patients made almost no suggestions for improvements regarding the videos. However, they raised 3 main concerns. First, 6 patients noted that not all individuals would identify with the content, stating, for example, “Social anxiety is so different for each person” or “If I do not experience my anxiety the way the video presents it, will I begin to think I do not have the disorder?” By contrast, 1 patient reflected on the risk of “self-diagnosis” that viewers might engage in while watching the videos. Second, 3 participants expressed frustration that the videos “only presented problems” and did not offer ways to cope with the symptoms. These 3 patients emphasized the need for further clarification of the types of treatment “we could offer them” to manage the issues presented in the videos.

Third, 5 patients expressed concerns that the videos might be distressing for some viewers, especially those dealing with the issues presented. For example, 1 patient reported becoming aware of their heartbeat while watching. As the videos were intended to help individuals on the waiting list identify with the scenarios presented, we considered this feedback potentially concerning and followed up with additional questions. We asked how distressed they found the videos and whether they had personal experience with the mental disorders portrayed. Interestingly, none of the patients reported significant distress in the form of anxiety, although they speculated that someone else might find the videos more distressing. The quantitative data corroborate these findings. Moreover, patients who spontaneously reported previous experiences with social anxiety or panic attacks rated these videos more highly than the other videos.

One patient reported difficulty concentrating during the 2 videos that presented clinical vignettes (only 2 were viewed due to a malfunction with the depression video) and described the sound design as chaotic. The same patient rated the videos as uncomfortable or offensive “to a very large degree.” In a follow-up question—“This is very important to us. We want to know what about the videos we made caused you to feel uncomfortable, and in what way.”—the patient explained that their discomfort stemmed from frustration at being unable to concentrate during the videos, rather than from anxiety or indignation. When asked further, the patient stated that they had no issues with the video on treatment options.

### Addressing the Usability Test Results in the Project Group

#### Issues Identified As Important

The project group discussed all major themes and the usability issues identified. The most pressing concern was the navigation issue, which made it difficult for patients to locate completed tasks in MyHealth outside the StartHelp dashboard. Most of the discussion focused on how to resolve these issues without modifying the MyHealth infrastructure, which would require changes deeper in the technological architecture. After reviewing the information presented to patients, the project group concluded that the guidance on how to schedule the first session as a video consultation required improvement. Other issues discussed included potential discomfort when watching the videos and whether they effectively served their intended purpose. Regarding the MyHealth app, an issue raised by user representatives during preliminary testing of the software following the paper prototype tests was that they experienced the login procedures as unnecessarily cumbersome.

We communicated all findings to the owners of MyHealth, which is part of the regional hospital administration, through 2 complementary approaches. First, we held verbal discussions to explore potential solutions that could be implemented without significantly altering MyHealth’s core features. Second, any new programs or modifications to existing programs require final approval from the owners—a safety mechanism that ensures appropriate regulation of MyHealth as a platform for innovation. Therefore, all requested changes were formally registered and archived.

#### Changes Made to MyHealth and StartHelp

Few changes were made to MyHealth during the development and usability testing of StartHelp. However, new login alternatives were added, which now include login via fingerprint or facial recognition, as well as faster login using a personalized numeric PIN (personal identification number). Following the usability testing of StartHelp, 1 improvement implemented was the provision of clearer information on how patients can request a video consultation for their first appointment. This information is now prominently displayed on the web page containing more in-depth details about the first session. We did not modify the videos themselves, as the treatment options video already presents video consultations as an option and encourages viewers to consult the additional information on the website. Notably, it was this video that prompted 1 patient to request more concrete information on the topic. Additionally, information on how to locate previously completed tasks is now clearly presented in bold at the bottom of every task: “Remember that all completed tasks can be found in the MyHealth menu under ‘Education’ and then ‘StartHelp.’ Go there if you wish to view your previous tasks.” Moreover, we updated the app to allow tasks to remain on the dashboard for a full week after completion.

Regarding the videos, we concluded that they were unlikely to cause unnecessary discomfort. One patient rated the 3 videos containing patient stories as uncomfortable or offensive “to a very large degree,” primarily due to chaotic sound and graphic design that made it difficult to concentrate. This patient did not report any issues with the treatment options video, which had been revised based on workshop feedback.

The project group reasoned that individuals who have difficulty tolerating the current video design are unlikely to be part of the target population for the associated treatments, which focus specifically on social anxiety disorder, panic disorder, or depression without concurrent disorders. Additionally, the videos are optional, presented alongside written information, and accessed primarily by users seeking in-depth information about these treatment modalities. Although some degree of emotional discomfort may be unavoidable when videos encourage patients to identify with patient stories, the overall reported discomfort during testing was relatively low.

Keeping StartHelp’s goal in mind—namely, to inform patients and encourage active involvement in treatment decisions so that they can better tailor their treatment—we chose not to modify the videos themselves. Instead, we revised the surrounding text to strengthen the connection between the videos and the clinic’s specific treatment options. For instance, using therapist-guided internet therapy as an example:

Panic disorder, social anxiety, and depression are three disorders currently being treated through therapist-guided internet therapy at the Centre for eTherapy. If you believe that one of these disorders may be your primary concern and are interested in trying out therapist-guided internet therapy, here is how to contact us for an assessment to determine whether this approach may be beneficial for you. Therapist-guided internet therapy typically has much shorter waiting lists, and participating in this assessment will not remove you from the current waiting list for other treatments.

We concluded that the aforementioned changes were sufficient. As the project group did not anticipate any new critical usability issues arising from the proposed changes, no additional isolated usability testing was deemed necessary. The remaining potential issues were considered best evaluated within the context of a clinical trial.

## Discussion

### Principal Findings

This study had 2 objectives: to describe the development of StartHelp and to explore its usability and perceived user satisfaction. The purpose was to evaluate whether StartHelp was ready for further evaluation in a clinical trial. The study, therefore, represents an exploratory, early-stage evaluation rather than definitive evidence of StartHelp’s usability or user satisfaction, as such evidence would require further testing in its intended real-world context. In the following sections, we discuss the development process in accordance with the GRIPP2 guidelines [19], the usability findings, user satisfaction with the video content, and implications for future research.

### Development Process

When developing StartHelp, we aimed to ensure that user representatives were involved as “partners” [33]. To achieve this, we engaged user representatives who had prior experience working with members of the project team and were familiar with the interventions on which StartHelp is based [7-9]. The project was designed with a long-term collaborative relationship in mind, reinforcing the academic team’s commitment to the perspectives and goals of user representatives. Our approach was informed by previous research on user involvement, which indicates that even in nonhierarchical collaborations, an indirect hierarchy can develop, especially when partnerships are short term [20]. However, partnering with the same user representatives over an extended period may also have drawbacks, as it could limit the diversity of viewpoints represented.

To mitigate this, we included patients with no prior experience working with the project group in the initial focus groups and subsequent usability testing. Another way to mitigate this issue could have been to involve at least one patient with no prior experience working with the project group. During the development phase, before usability testing, we primarily involved patients with attention-deficit/hyperactivity disorder or autism spectrum disorder, which may have introduced bias. As previously stated, however, we reasoned that if StartHelp is accessible to these populations, it should also be accessible to most other populations.

### Usability

In line with the recommendations of Maramba et al [36], the usability evaluation included both quantitative and qualitative data. The quantitative data revealed some discrepancies. The average SUS scores for the sample were 63.6 for the MyHealth app and 83.7 for the videos. Notably, none of the patients rated the MyHealth app higher than the videos, indicating that navigating the overarching MyHealth app was more complicated than engaging with the informational video content and using the StartHelp dashboard. According to Bangor et al [47], a score of 83.7 is considered good to excellent, whereas a score of 63.7 is deemed acceptable. Moreover, a mean score of 63.6 is below the benchmark for digital health apps (68) [39] and for other Norwegian patient portals (72.1) [28]. This suggests that MyHealth’s usability may be lower than that of the average health app or patient portal. Intuitively, there should not be such a large difference in SUS scores between the Norwegian benchmark values and those observed for MyHealth. This discrepancy may be explained either by the small sample size or by interactions between StartHelp’s features and MyHealth, which may result in usability challenges not present when using MyHealth alone. This finding is explored further below in the discussion of the qualitative data. However, the marked difference between usability ratings for StartHelp and the MyHealth portal further suggests that participants were able to distinguish between the 2 systems during testing.

### Satisfaction and Video Quality

Mean scores and SDs on the CSQ-4 are comparable to those observed in Norwegian specialist mental health care [42,48,49], suggesting that the current sample was representative in this regard. Moreover, none of the patients who participated in the usability testing reported extreme CSQ-4 scores before testing, making it less likely that overall treatment satisfaction biased the results. By contrast, scores on the CSQ-Video indicated satisfaction in the lower medium range [43]. Few studies have used the CSQ in the context of pretherapy interventions [8,50], and only 1 used the CSQ to evaluate patients’ satisfaction with the intervention itself [8]. By contrast, Zwick and Attkisson [50] used it to assess how their intervention affected patients’ perceptions of services provided after treatment ended. Lara-Cabrera et al [8] reported an item-level mean of 3.28 on the CSQ-8 [8], which is higher than the item-level mean in this study (2.73). This discrepancy may be attributed to the use of different CSQ versions; however, this explanation seems unlikely because the CSQ-8 and CSQ-4 have demonstrated similar psychometric properties and mean values in outpatients receiving specialized mental health care [42,51]. The discrepancy is more likely related to CSQ’s association with respondents’ personal needs at the time of assessment [44,52] or to the expected clinical utility within the study context. We would not expect the brief, nonclinical context of this study’s usability testing to be sufficient to address participants’ current problems in a general sense (ie, to alleviate mental health symptoms). This interpretation is further supported by the reported perceived quality of the videos, which was considerably higher than the CSQ-Video scores. For example, patient 5 reported low satisfaction (10; cutoff 10.5 [49]) yet rated the video quality as high (mean score 92.5 out of 100). Nevertheless, further research on the full StartHelp intervention—tested in its intended context, in which patients awaiting treatment receive the complete program—is needed to more comprehensively evaluate user satisfaction with the program.

### Qualitative Findings

In general, the qualitative data derived from patients’ task completion and responses to open-ended questions corroborated the quantitative findings. Most reported usability problems stemmed from attributes of the MyHealth app (eg, default language settings and text size) and from its navigation. Patients reported no usability issues with the StartHelp dashboard. Other researchers have noted that a major barrier to the widespread integration and implementation of digital interventions is poor or outdated information technology infrastructure [16,53,54]. Our results may reflect a similar tension between the infrastructure and the specific interventions designed within it. Building apps within an overarching app and patient portal, such as MyHealth, offers several advantages, including secure handling of patient data and integration with the patient administration system to provide easy access to other health information collected through routine clinical practice. One downside, however, is that app development within such infrastructures may be constrained in terms of design and available features. Moreover, adding new features to an app or intervention may prove difficult if doing so requires fundamental changes to the overarching technological infrastructure. It is also challenging to predict whether such modifications will result in unintended interactions with other functions or integrated apps. We attempted to mitigate these challenges by conducting initial prototype testing and limiting changes to MyHealth, instead modifying only aspects of StartHelp, as discussed above in the “Changes Made to MyHealth and StartHelp” section. These changes were intended to minimize the need for patients to switch between the MyHealth menu and the StartHelp dashboard.

Patients also offered reflections on the videos, which primarily fell into 2 categories: their function and their potential to cause discomfort. Ultimately, we modified the accompanying information rather than the videos themselves. Interestingly, although several patients expressed concern that the videos might disturb others and provoke anxiety symptoms, they reported experiencing few symptoms themselves. Although we did not alter the videos, we ensured that none were mandatory and that all content was also readily available in text format.

### Strengths and Limitations

Five users are often sufficient to identify approximately 80% of usability problems [35]. However, this depends largely on the nature and number of issues present in the program; common and critical problems are generally easier to detect. Although we cannot be certain that all usability issues were identified, most of the tasks assigned to patients were completed without difficulty. Therefore, we consider our sample size of 10 adequate for identifying critical usability issues before further testing in a clinical trial. A strength of the study is that actual patients were involved in the testing and that both quantitative and qualitative data were collected [36]. However, a sample of 10 is small for quantitative estimates of usability and satisfaction, and further evaluation with larger samples is required. The qualitative data consisted primarily of written responses to open-ended questions; in-depth interviews could provide additional insights.

Another strength is the involvement of user representatives, as well as the study’s novelty. To our knowledge, no other digital pretherapy interventions have been developed for a general outpatient population in specialized mental health care in close collaboration with user representatives.

Although sex was evenly distributed in our sample, most participants were highly educated and relatively young. We consider the sample representative of the clinical population treated at the outpatient clinic and at other similar Norwegian outpatient specialized mental health services [55]. Nevertheless, the absence of older adults or individuals with little formal education is a limitation. Although these groups represent minorities in similar outpatient settings [53], further real-world testing should examine whether they encounter difficulties using StartHelp—for example, by assessing lower app usage or higher dropout rates. As we developed StartHelp for a Norwegian context, the results may not be generalizable to international settings. However, some issues that emerged during testing may be transferable, as developing programs within existing infrastructures may be necessary for full implementation within current health care information technology systems. Moreover, MyHealth is comparable to other patient portals across Norway and other Nordic countries due to collaborations with the same health system providers [35,37,38].

Another limitation is the staged setting in which the testing took place. We assessed the usability of the program itself; however, we do not know whether issues specific to more naturalistic use may arise during a potential feasibility trial involving a waiting list population, in which most users are unlikely to explore all aspects of the app simultaneously. The current study design is well-suited to identifying concrete usability and technical issues related to navigating and using StartHelp. However, further research is needed to evaluate how patients use StartHelp before their first consultation and how this influences patient satisfaction.

Additionally, further research is needed to establish StartHelp’s usability and to evaluate its preliminary effects on patient satisfaction with treatment. Conducting a randomized controlled feasibility trial that recruits patients awaiting their first consultation in specialized mental health care would be particularly valuable. Moreover, StartHelp’s intended downstream effects on treatment outcomes—by enhancing treatment satisfaction and patient-reported outcomes associated with improved clinical outcomes—should be investigated further.

### Conclusions

Through a collaborative co-creation process, we developed StartHelp, a pretherapy psychoeducation program for outpatients awaiting specialized mental health treatment. During usability testing, a few issues related to StartHelp itself emerged; however, participants reported difficulties navigating the overarching MyHealth portal, which received usability scores lower than hypothesized. The project group discussed and addressed the problems identified during usability testing, thereby minimizing the need to navigate the overarching patient portal. Although the perceived quality and usability of the clinical material were high, patients reported lower satisfaction than anticipated. Taken together, these findings suggest that StartHelp is suitable for further evaluation in a feasibility trial conducted within its intended context, involving outpatients awaiting specialized mental health treatment.

## Appendix

Multimedia Appendix 1 The Guidance for Reporting Involvement of Patients and the Public (GRIPP2) checklist.

Multimedia Appendix 2 Treatment options mentioned by patients during usability testing.

Multimedia Appendix 3 Post-it notes created during prototype testing and the abstracted themes.

## Abbreviations

CSQ-4: 4-item Client Satisfaction Questionnaire
CSQ-Video: a modified version of the Client Satisfaction Questionnaire 4-item version
GRIPP2: Guidance for Reporting Involvement of Patients and the Public
PIN: personal identification number
SIKT: Kunnskapssektorens tjenesteleverandør (Norwegian Agency for Shared Services in Education and Research)
SUS: System Usability Scale
