# Supplementary material for: Co-Created Digital Pretherapy Psychoeducation for Outpatients in Specialized Mental Health Care: Usability Evaluation and Patient Satisfaction Study
Source: JMIR Hum Factors. 2026 Feb 26;13:e80130. doi: 10.2196/80130 (PMC12982959; doi:10.2196/80130)
Supplement: Multimedia Appendix 1 [file humanfactors_v13i1e80130_app1.pdf]

Section and Topic	Item	Description	Reported on Page No.
Section 1: Abstract of paper	1a	Report the aim of the study	2
	1b	Describe the methods used by which patients and the public were involved	2
	1c	Report the impacts and outcomes of PPI in the study	2
	1d	Summarise the main conclusions of the study	2-3
	1e	Include PPI, “patient and public involvement,” or alternative terms as keywords	3
Section 2: Background to paper	2a	Report the definition of PPI used in the study and how it links to comparable studies	5
	2b	Report the theoretical rationale and any theoretical influences relating to PPI in the study	5
	2c	Report any conceptual models or influences used in the study	5
Section 3: Aims of paper	3	Report the aim of the study	6
Section 4: Methods of paper	4a	Provide a clear description of methods by which patients and the public were involved	9-11, 29
	4b	Provide a description of patients, carers, and the public involved with the PPI activity in the study	8-9
	4c	Report on how PPI is used at different stages of the study	9-11, 29
	4d	Report the level or nature of PPI used at various stages of the study	5, 8-9
Section 5: Capture or measurement of PPI impact	5a	If applicable, report the methods used to qualitatively explore the impact of PPI in the study	Not applicable
	5b	If applicable, report the methods used to quantitatively measure or assess the impact of PPI	Not applicable
	5c	If applicable, report the rigour of the method used to capture or measure the impact of PPI	Not applicable
Section 6: Economic assessment	6	If applicable, report the method used for an economic assessment of PPI	Not applicable
Section 7: Study results	7a	Report the results of PPI in the study, including both positive and negative outcomes	Not applicable
	7b	Report the positive and negative impacts that PPI has had on the research, the individuals involved (including patients and researchers), and wider impacts	Not applicable
	7c	Report the influence of any contextual factors that enabled or hindered the process or impact of PPI	Not applicable
	7d	Report the influence of any process factors that enabled or hindered the impact of PPI	Not applicable

	7e,i	Report any conceptual or theoretical development in PPI that have emerged	Not applicable
	7e,ii	Report evaluation of theoretical models, if any	Not applicable
	7f	If applicable, report all aspects of instrument development and testing (e.g., validity, reliability, feasibility, acceptability, responsiveness, interpretability, appropriateness, precision)	Not applicable
	7g	Report any information on the costs or benefit of PPI	Not applicable
Section 8: Discussion and conclusions	8a	Comment on how PPI influenced the study overall. Describe positive and negative effects	32
	8b	Comment on the different impacts of PPI identified in this study and how they contribute to new knowledge	8-10, 17-19
	8c	Comment on the definition of PPI used (reported in the Background section) and whether or not you would suggest any changes	8
	8d	Comment on any way your study adds to the theoretical development of PPI<br><br>*Not applicable. This study aimed to use PPI as a tool, not develop or study PPI itself.*	Not applicable
	8e	Comment on how context factors influenced PPI in the study	32
	8f	Comment on how process factors influenced PPI in the study	32
	8g	If applicable, comment on how well PPI impact was evaluated or measured in the study	Not applicable
	8h	If applicable, discuss any aspects of the economic cost or benefit of PPI, particularly any suggestions for future economic modelling	Not applicable
	8i	Comment critically on the study, reflecting on the things that went well and those that did not, so that others can learn from this study	32
