# Supplementary material for: Co-Created Digital Pretherapy Psychoeducation for Outpatients in Specialized Mental Health Care: Usability Evaluation and Patient Satisfaction Study
Source: JMIR Hum Factors. 2026 Feb 26;13:e80130. doi: 10.2196/80130 (PMC12982959; doi:10.2196/80130)
Supplement: Multimedia Appendix 2 [file humanfactors_v13i1e80130_app2.pdf]

Patient 1

Before testing:

Individual

Group

Internet

After testing:

Individual

Group

Talking over Video

Patient 2

Before testing:

No suggestions

After testing:

Group

Individual

Video consultations

Medication

Patient 3

Before testing:

no suggestions

After testing:

Group

Video consultations

Face to face psychotherapy

Patient 4

Before testing:

Talking therapy

Group therapy

Online talking with psychologist

After testing:

Physical therapy

Internet treatment

Medical treatment

Individual talking with psychologist

Group treatment

Patient 5

Before testing:

Consultations

Group therapy

Internet therapy

After testing:

Consultations

Group therapy

Medications

Patient 6

Before testing:

Talking with psychologist

Diagnostic courses and education

After testing:

Talking with psychologist

Diagnostic courses and education

Patient 7

Before testing:

No suggestions

After testing:

Talking to psychologist

Group treatment

Patient 8

Before testing:

No suggestions

After testing:

No suggestions

Patient 9

Before testing:

Psychiatry/psychologist

Physical therapy

Group exposure therapy

Counselling by vocational counsellor

Family psychoeducation

After testing:

Psychiatry/psychologist

Physical therapy

Group exposure therapy

Counselling by vocational counsellor

Family psychoeducation

Patient 10

Before testing:

Psychoeducation

Group consultation

Psychologist talking

After testing:

Psychoeducation

Group consultation

Psychologist talking
