# Supplementary material for: Co-Created Digital Pretherapy Psychoeducation for Outpatients in Specialized Mental Health Care: Usability Evaluation and Patient Satisfaction Study
Source: JMIR Hum Factors. 2026 Feb 26;13:e80130. doi: 10.2196/80130 (PMC12982959; doi:10.2196/80130)
Supplement: Multimedia Appendix 3 [file humanfactors_v13i1e80130_app3.pdf]

Figure S1. Visual representation of comments made by users in prototype testing

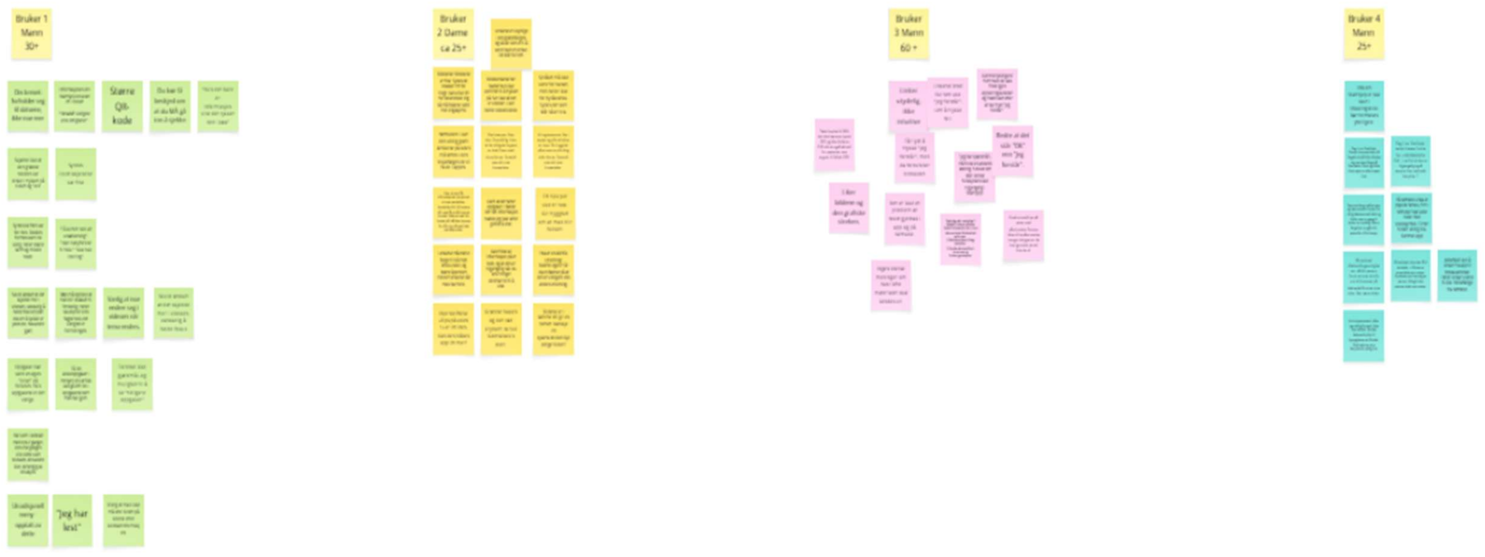

Figure S2. Visual representation of themes emerging from prototype testing

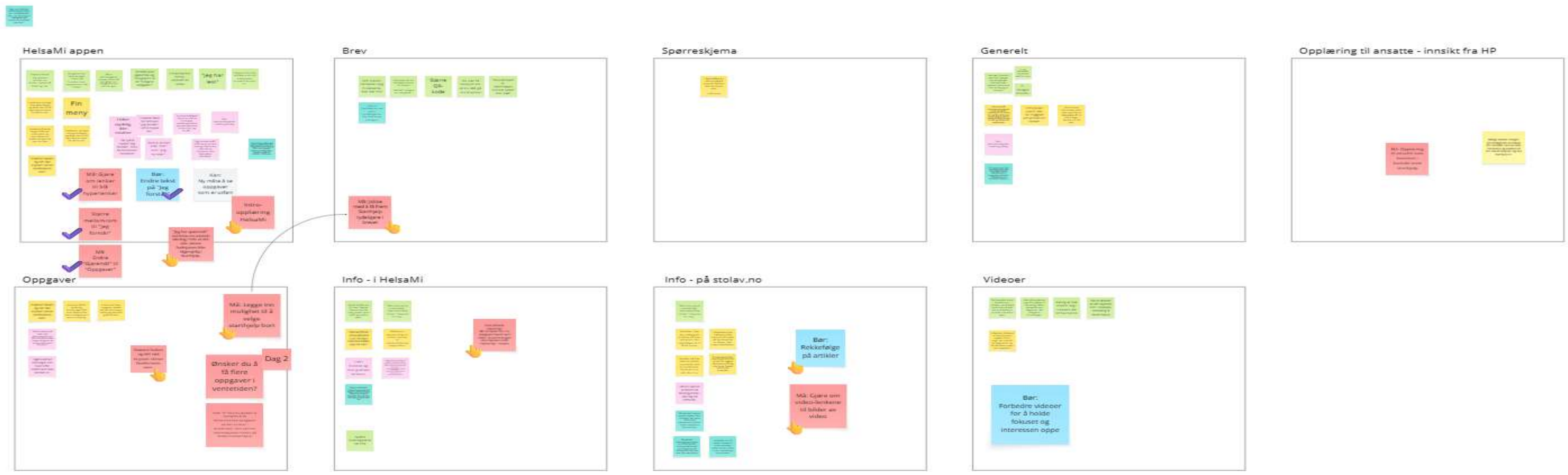

HelsaMi appen
"Jeg har lest"
Fin meny
Bør: Endre tekst på "Jeg forstår"
Intro-opplæring HelsaMi
Oppgaver
Må: Legge inn mulighet til å velge starthjelp tidlig
Ønsker du å få flere oppgaver i ventetiden?
Dag 2
Brev
Samme QR-kode
Spørreskjema
Generelt
Opplæring til ansatte - innsikt fra HP
Info - i HelsaMi
Info - på stolav.no
Bør: Rekkefølge på artikler
Må: Gjøre om video-lenkene til bilder av video
Videoer
Bør: Forbedre videoer for å holde fokuset og interessen oppe
